# Aromatic effects of a Japanese citrus fruit—yuzu (*Citrus junos* Sieb. ex Tanaka)—on psychoemotional states and autonomic nervous system activity during the menstrual cycle: a single-blind randomized controlled crossover study

**DOI:** 10.1186/s13030-016-0063-7

**Published:** 2016-04-21

**Authors:** Tamaki Matsumoto, Tetsuya Kimura, Tatsuya Hayashi

**Affiliations:** Department of Health Education, Faculty of Education, Shitennoji University, 3-2-1 Gakuenmae, Habikino, Osaka 583-8501 Japan; Graduate School of Human Development and Environment, Kobe University, Hyogo, Japan; Cognitive and Behavioral Science, Graduate School of Human and Environmental Studies, Kyoto University, Kyoto, Japan

**Keywords:** Yuzu, Olfactory stimulation, Heart rate variability, Autonomic nervous system activity, Menstrual cycle, Profile of mood states

## Abstract

**Background:**

Yuzu (*Citrus junos* Sieb. ex Tanaka), a yellow-golden colored citrus fruit, has traditionally been used to promote psychosomatic health in Japan. While the yuzu produces a distinctive, pleasing aroma of citrus and floral, the efficacy of its fragrance remains unknown. The present study investigated the soothing effects of the fragrance of yuzu essential oil from the perspective of autonomic nervous system activity, which plays a crucial role in the integrity of the mind-body connection.

**Methods:**

Twenty one women in their 20s participated in a single-blind randomized controlled crossover study. Subjects were examined twice each in the follicular and late-luteal phases. Two kinds of aromatic stimulation (yuzu and water as a control) were used. This experiment measured heart rate variability (HRV) reflecting autonomic nervous system activity and used the Profile of Mood States (POMS) as a psychological index before and after the aromatic stimulation.

**Results:**

Only a 10-min inhalation of the yuzu scent significantly decreased heart rate and increased high frequency power of HRV reflecting parasympathetic nervous system activity, regardless of menstrual phase. This significant physiological effect continued for at least 25 min. In addition, the POMS tests revealed that inhalation of the aromatic yuzu oil significantly decreased total mood disturbance, a global measure of affective state, together with two POMS subscales—tension-anxiety and fatigue, as long as 35 min after the aroma stimulation, both in the symptomatic late-luteal and non-symptomatic follicular phases.

**Conclusions:**

The present study provides the novel information that yuzu’s aromatic effects could serve to alleviate negative emotional stress, which, at least in part, would contribute to the improvement of parasympathetic nervous system activity.

## Background

According to the systemic investigation of national mental health surveys, stress-related psychological and emotional disorders are 20 to 40% more common in women than men in any given year [1]. As to stress management strategies, women often seek safe and effective options for their healthcare conditions. The practice of aromatherapy—a form of complementary and alternative medicine—uses volatile plant materials, known as essential oils, to balance mind, body, and spirit and has a long history of use in supporting women’s health and lifestyles [2, 3]. A number of essential oils are currently in use as aromatherapy agents to relieve stress, anxiety, and depression. Popular oils include lavender, rose, orange, bergamot, lemon, sandalwood, clary sage, Roman chamomile, and rose-scented geranium [4].

Beyond its role as an essential ingredient in food products and various cuisines, yuzu (*Citrus junos* Sieb. ex Tanaka), a yellow-golden colored citrus fruit resembling a small orange or tangerine, has traditionally been used to promote mind and body health in Japan. For instance, taking a *yuzu-yu* (yuzu bath), a hot bath in which whole yuzu fruits are floated, is a winter solstice custom that dates back to at least the early 18th century. While enjoying the yuzu aroma, a relaxing soak relieves stress and creates a feeling of well-being. The yuzu bath is also said to warm the body, guard against colds, and treat arthritis, rheumatism, and the roughness of skin [5]. The essence of yuzu fragrance falls somewhere between grapefruit and mandarin with subtle overtones of bergamot and lime, while producing a very appealing, almost floral note [6]. Because of its distinctive pleasing fragrance, aromatic and/or cosmetic products of yuzu are now commercially available as a healthcare modality. Studies from Japanese scientific journals suggest the healing effects of the yuzu fragrance and its potential application to aromatherapy [7, 8]. However, an extensive literature search for the present study, using the PubMed database, revealed a paucity of empirical human-subject research regarding the efficacy of yuzu fragrance on the psychophysiological health for women.

The autonomic nervous system plays vital roles in dynamically controlling the response of the body to a range of external and internal stimuli and ingeniously modulating biological homeostasis in humans. Instability, or even a slight disorder of the system, therefore, could induce broadly ranged psychophysiological phenomena and, ultimately, far-reaching adverse effects on health. An electrocardiogram (ECG) sampling of R-R interval variation is regulated by the net effect of sympathetic and parasympathetic input. Comprehensive and functional analysis of heart rate variability (HRV) has served as a reliable noninvasive technique for quantitative assessment of cardiovascular autonomic regulatory responses, providing a dynamic map of sympathetic and parasympathetic interaction [9]. Clinical studies have consistently shown reduced HRV in people with work stress [10] or psychosomatic symptoms such as depression [11], anxiety [12], and chronic fatigue syndrome [13]. A series of the authors’ previous research has shown that autonomic function, evaluated by HRV, was altered among women with psychosomatic problems, including premenstrual [14, 15] or menopausal symptoms [16]. In contrast, individuals with greater emotion-regulation ability have been shown to have greater levels of resting HRV [17]. Several studies have investigated clinical benefits of aromatherapy by using HRV and demonstrated significant changes of autonomic nervous system activity after inhalation of essential oils, including lavender [18–21], jasmine [19] and bergamot [22–24], while affecting mood, mental conditions and behavior. These findings demonstrate the functionality of HRV measurements to evaluate psychophysiological effects of aromatherapy with yuzu fragrance from the perspective of autonomic nervous system activity as it reflects mind and body interaction.

Accordingly, the present study used a single-blind randomized controlled crossover design to measure the effects of commercially available yuzu essential oil on mood states and autonomic nervous system activity by using HRV measurements in the follicular and luteal phases of eumenorrheic women. A majority of women experience a regular recurrence of various symptoms in the premenstrual phase, although the symptom complex does not always reach clinically significant levels, such as premenstrual syndrome (PMS) or premenstrual dysphoric disorder (PMDD) [25]. This study then investigated if inhalation of yuzu fragrance could serve as an additional modality to alleviate psychoemotional stress and if the menstrual cycle influences the efficacy of yuzu aromatherapy among reproductive-age women.

## Methods

### Subjects

Twenty one women in their 20s volunteered to participate in a single-blind randomized controlled crossover study. The women, all college students, responded to a campus advertisement. The study protocol was approved in advance by the Institutional Review Board of Shitennoji University and was performed in accordance with the Declaration of Helsinki of the World Medical Association. All subjects received an explanation of the nature and purpose of the study: to investigate soothing effects of plant fragrance on emotional symptoms during the menstrual cycle. We did not, however, inform subjects of which fragrance we would use for the experiments. We mentioned neither the concentration of the fragrance nor the use of water as a control trial. Prior to receiving any data about the experiments, all subjects gave their written informed consent to participate in the study.

The subjects underwent medical examinations and interviews and completed a standardized health questionnaire regarding medical history, medications, current health condition, regularity of menstrual cycle, premenstrual discomfort, and lifestyle. While referring to subjects’ self-reported regular menstrual cycles, we determined the cycle phase during the experiments by the onset of menstruation and oral temperature and verified it by the concentrations of ovarian hormones, estrone (E1), and pregnanediol-3-glucuronide (PdG), in a urine sample taken early in the morning. Both E1 and PdG were indexed to creatinine (Cr) excretion [14, 15, 26].

Menstrual-cycle related discomfort, such as PMS and PMDD, influences the autonomic nervous system [14, 15, 26–28]. As in our previous studies [14, 15], therefore, we asked subjects to answer the menstrual distress questionnaire [29] in the follicular (the fifth to the eleventh day from the first day of menstruation) and the late-luteal phase (within seven days before the next menstruation) to determine the severity of premenstrual symptoms. The average value of the increase on the total scores of the questionnaire from the follicular to the late-luteal phase was 4.0 ± 2.5 %. None of the women reported that premenstrual symptoms markedly interfered with work, school, usual activities or relationships with others. Considering this information, no subjects in the present study suffered from PMS or PMDD, but the subjects’ symptoms did fall within the sphere of premenstrual molimina, signalling impending normal menstruation, which a majority of reproductive-age women experience [25, 30].

None of the subjects had been clinically diagnosed with diabetes mellitus, hypertension, hyperlipidemia, or cardiovascular or any other endocrine or systemic disorders that could affect the autonomic nervous system. The subjects were non-obese and non-smokers. Before starting the study, none of the women reported taking oral contraceptives to control the menstrual cycle or any other medications influencing the autonomic nervous system.

Referring to human-subject research on olfactory stimulation from aroma [6, 21, 31], this study performed the olfactory function test on all subjects to assure that none had anosmia. Briefly, subjects were given two sets of three bottles—two held distilled water; the third contained essential oils (yuzu or lavender)—and were asked to choose the one that differed from the other two. To be eligible for the study, subjects had to choose the correct response in both trials.

All subjects were asked not to consume any food or beverages containing alcohol or caffeine after 21:00 of the day preceding the experiment. The subjects were also instructed to abstain from alcohol use and excessive physical activity for 24 h before testing [14, 21].

### Experimental procedure

Subjects were examined twice each (aroma and control trials) in the follicular (the fifth to the eleventh day from the first day of menstruation) and late-luteal phases (within seven days before the next menstruation). Considering subjects’ menstrual cycles and availability to come to the laboratory for this research project, the order of testing was randomized. Table 1 summarizes the randomization of four trials. As the table indicates, for instance, Subject 1 participated in the trials in the following order: Late-luteal yuzu, follicular yuzu, late-luteal water, follicular water trials. Two subjects—S3 and S5—discontinued the trials due to menstrual irregularity (extended menstrual duration) and taking a prescribed medication, during the study, respectively. One subject (S8) could not manage her schedule to participate in the remaining three trials after the late-luteal water trial. Therefore, 18 subjects completed all four trials.

**Table 1:** Randomization of four trials

	Follicular		Late luteal	
Subject number	Yuzu	Water	Yuzu	Water
S1	2	4	1	3
S2	2	3	1	4
S3	2	1		
S4	2	4	1	3
S5			1	2
S6	4	3	2	1
S7	4	1	2	3
S8				1
S9	3	1	2	4
S10	2	3	1	4
S11	3	2	4	1
S12	3	4	1	2
S13	3	4	1	2
S14	2	1	3	4
S15	2	3	1	4
S16	1	4	3	2
S17	3	1	2	4
S18	1	2	3	4
S19	3	4	2	1
S20	1	4	3	2
S21	1	2	3	4

The numbers (1, 2, 3, and 4) indicate the order of trials each subject participated in

All measurements were taken between 11:00 and 15:00 and were performed in a temperature-controlled (25 °C), quiet, comfortable room with a minimization of arousal stimuli. Height and body weight of each subject were measured to calculate body mass index (BMI) as body weight divided by height squared. Subjects then rested for at least 10 min before the start of the experiment.

This experiment used two kinds of aroma stimulation: yuzu (*Citrus junos* Sieb. ex Tanaka, Lot No. 20, TREE OF LIFE Co., Ltd., Tokyo, Japan) and water as a control. The major components of the yuzu essential oil used in this study are as follows: limonene 78.02 %, γ-terpinene 9.32 %, β-myrcene 1.77 %, α-pinene 1.34 %, δ-elemene 0.79 %, β-pinene 0.69 %, β-caryophyllene 0.60 %, α-phellandrene 0.43 %, terpinolene 0.41 %, p-cymene 0.38 %, α-terpinenol 0.07 %, linalool 0.03 % (unidentified minor compounds constitute the remaining 6.15 %).

According to previous research on aromatherapy with floral and citrus fruit essential oil [6, 21], we pipetted 10 μl of yuzu essential oil or water onto a small cotton pad designed for a diffuser (Aroma breeze NOVA T, ALTA Corporation, Nagoya, Japan). Airflow from the diffuser was set at 1.3 m per min and placed near the subject’s nostrils using the diffuser’s 30-cm long circular cylinder fitted with a perforated funnel (diameter 5 cm).

Before measurements were taken, the subjects were instructed to relax quietly and comfortably for at least 10 min in a seated position while equipped with electrocardiograph (ECG) electrodes. They then filled out the Profile of Mood States (POMS) explained in detail below. The ECG was recorded 5 min before inhalation of the scent. Each subject then inhaled the scent for 10 min. Right after the aroma stimulation, each subject rated the scent’s intensity, pleasantness and familiarity, and her likes and dislikes regarding the scent by using a visual analogue scale (VAS) with a 10-cm horizontal line [31]. We again measured the ECG for 5 min at 0, 10, 20, and 30 min after inhalation. During ECG recording, all subjects breathed in synchrony to a metronome at 15 beats per minute to ensure that the respiratory-linked variations in heart rate did not overlap with low-frequency heart-rate fluctuations (below 0.15 Hz) from other sources [15, 21]. After the ECG was recorded, the subjects repeated the POMS test. The ECG signals were later analysed by means of HRV power spectral analysis, as described below, to evaluate whether aroma stimulation changed autonomic nervous system activity.

### Evaluation of autonomic nervous system activity

The technique of the HRV power spectral analysis for the present investigation has been applied in basic physiological and clinical research fields, and its validity and reliability have been previously confirmed [15, 21, 32]. We thus briefly explain the procedure of R-R interval power spectral analysis used in the present study. The ECG signal was amplified (MEG-6108, Nihon Kohden, Tokyo, Japan) and digitized via a 16-bit analog-to-digital converter (Model PS-2032GP, TEAC, Tokyo, Japan) at a sampling rate of 1000 Hz. The digitized ECG signal was differentiated, and the resultant QRS spikes and the intervals of the impulses (R-R intervals) were stored sequentially on a hard disk for later analysis.

Before the R-R spectral analysis was performed, the stored R-R interval data were aligned sequentially to obtain equally spaced samples with an effective sampling frequency of 2 Hz and displayed on a computer screen for visual inspection. Then, the direct current component and linear trend were completely eliminated by digital filtering for the band-pass between 0.03 and 0.5 Hz. The root mean square value of the R-R interval was calculated as representing the average amplitude. After passing through the Hamming window, power spectral analysis by means of a fast Fourier transform was performed on a consecutive 256-s time series of R-R interval data obtained during the test. Spectral powers were calculated for the following respective frequency band**s**: low frequency (LF) power (0.03–0.15 Hz), an indicator of both sympathetic and parasympathetic nervous system activity; high frequency (HF) power (0.15–0.5 Hz), which solely reflects parasympathetic nerve activity; and total power (0.03–0.5 Hz) representing overall autonomic nervous system activity.

Basal heart rates and autonomic nervous system activities differ from individual to individual. Thus, the mean values for heart rates before inhalation of a scent were set as the baseline values and the mean values for autonomic nervous system activity before the inhalation were standardized to 100 %. The rate of change after the inhalation was compared between aroma and control trials [19, 21].

### Assessment of emotional symptoms

We administered the Japanese version of the POMS test (Kaneko Shobo Co., Tokyo, Japan), a globally standardized, self-administered, 65-item questionnaire (including 7 dummy items) to assess mood states before and after inhalation of the yuzu aroma and water. Participants rated each item in terms of how strongly they had felt it during the past week, including the current day, on a five-point Likert-type scale of zero to four, ranging from “not at all” to “extremely.” We added these raw scores to generate six subscales of emotional state: tension–anxiety, depression–dejection, anger–hostility, vigor, fatigue, and confusion. These added raw scores were then converted into T-scores according to the POMS manual [33]. As a global measure of affective state, we also calculated a total mood disturbance (TMD) score, with higher scores indicating more mood disturbance, by adding the T-scores on the six subscales, with vigor negatively weighted [6, 21, 34]. It should be noted that the POMS test has been used to evaluate acute effects of clinical intervention including aromatherapy [6, 19, 21], since the scores reflect temporal moods and feelings that can change depending on a situation someone is in [33]. Referring to previous aroma research [6, 19, 21], to investigate the effect of the yuzu aroma on mood states, we compared changes in the POMS scores of the yuzu and control trials before and after the ECG measurements in the follicular and late-luteal phases.

### Statistical analysis

To investigate the influence of inhalation of the yuzu aroma on autonomic nervous system activity, we evaluated the effects of menstrual cycle, aroma, and time and their interactions (cycle x aroma, cycle x time, aroma x time, cycle x aroma x time) using three-way ANOVA with repeated measures. As to psychoemotional symptoms, we performed two-way ANOVA with repeated measures to evaluate the effects of menstrual-cycle and aroma and the interactions (cycle x aroma) on scores of the VAS scale rated right after the aroma inhalation period and changes in scores of the POMS test before and 35-min after the aroma inhalation period. Values are reported mean ± standard errors (SE). *P* values < 0.05 were considered statistically significant. All statistical analysis was performed using a commercial software package (IBM SPSS Statistics Version 22).

## Results

### Clinical characteristics of subjects

Table 2 represents the mean values of the physical features of 18 subjects who completed the aroma and water control trials in the follicular and late-luteal phases. The aroma and control experiments took place on the 7.6 ± 0.4th day and the 7.8 ± 0.4th day in the follicular phase and the 26.8 ± 0.6th day and the 27.6 ± 0.8th day in the late-luteal phase from the first day of menstruation, respectively. The interval between the two trials was 2.4 ± 0.3 days and 2.1 ± 0.3 days in the follicular and the late-luteal phases, respectively.

**Table 2:** Physical features of subjects (*n* = 18)

Age (years)	20.8 ± 0.2
Height (cm)	160.1 ± 1.4
Weight (kg)	52.8 ± 1.3
Body Mass Index (kg/m^2^)	20.6 ± 0.4
Menstrual cycle (days)	30.4 ± 0.9
Duration of menstrual flow (days)	6.6 ± 0.3

The values are given as means ± SE

To confirm regular ovulatory menstrual cycles among subjects, we measured their oral temperatures (MC-172 L, Omron, Kyoto, Japan) and urinary ovarian hormone concentrations in the follicular phase (7.5 ± 0.5th day) and in the late-luteal phase (26.0 ± 0.7th day). The basal body temperature in the late-luteal phase significantly increased from that of the follicular phase (36.54 ± 0.09 vs. 36.26 ± 0.05 °C, *P* = 0.001). We also found significant late-luteal increase in urinary ovarian hormones compared to the follicular phase (E1: 20.9 ± 2.5 vs. 10.5 ± 2.0 ng/ml Cr, *P* < 0.001; PdG: 1.59 ± 0.23 vs. 0.27 ± 0.05 μg/ml Cr, *P* < 0.001).

### Comparisons of acute psychological effects between yuzu and control trials

Table 3 shows the acute psychological effects evaluated with VAS right after the 10-min aroma stimulation with yuzu and water in the follicular and late-luteal phases. We found no menstrual-cycle effects and the interaction (cycle x aroma) on any items, but significant aroma effects on all four items—intensity, likes and dislikes, pleasantness, and familiarity.

**Table 3:** Comparison of acute psychological effects evaluated with the visual analogue scales right after the inhalation of yuzu or water

	Follicular		Late luteal		
	Yuzu	Water	Yuzu	Water	ANOVA
Intensity	6.1 ± 0.5	0.5 ± 0.3	6.5 ± 0.5	0.7 ± 0.3	Cycle effect: *F*(1,17) = 1.84, *P* = 0.19
					Aroma effect: *F*(1,17) = 118.30, *P =* <0.001^*^
					Interaction: *F*(1,17) = 0.15, *P* = 0.71
Likes and dislikes	8.1 ± 0.4	4.8 ± 0.3	8.5 ± 0.4	4.5 ± 0.3	Cycle effect: *F*(1,17) = 0.035, *P* = 0.85
					Aroma effect: *F*(1,17) = 67.37, *P =* <0.001^*^
					Interaction: *F*(1,17) = 2.68, *P* = 0.12
Pleasantness	7.5 ± 0.3	4.5 ± 0.4	7.6 ± 0.3	4.7 ± 0.3	Cycle effect: *F*(1,17) = 0.29, *P* = 0.60
					Aroma effect: *F*(1,17) = 44.93, *P =* <0.001^*^
					Interaction: *F*(1,17) = <0.001, *P* = 0.99
Familiarity	8.1 ± 0.3	4.8 ± 0.4	8.1 ± 0.3	4.9 ± 0.6	Cycle effect: *F*(1,17) = 0.012, *P* = 0.91
					Aroma effect: *F*(1,17) = 68.39, *P =* <0.001^*^
					Interaction: *F*(1,17) = 0.049, *P* = 0.83

The values are given as means ± SE; ^*^Statistical significance

### Autonomic nervous system activity before and after inhaling aromas

**Fig. 1:**
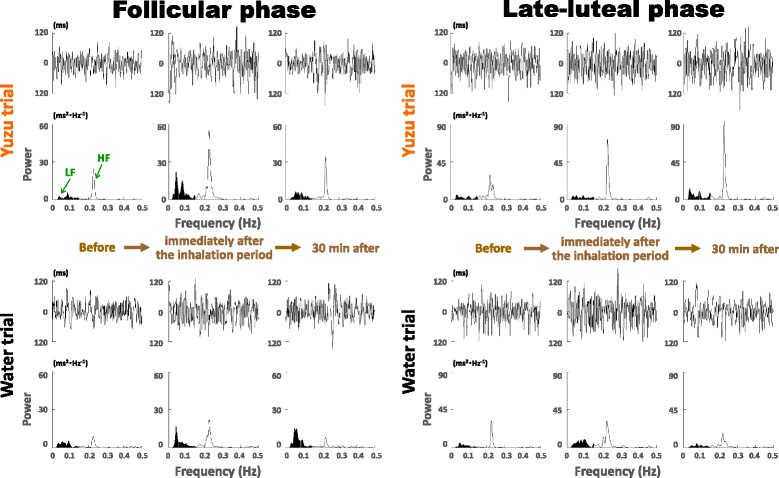
ECG R-R interval changes and corresponding power spectra before, immediately after, and 30 min after the 10-min inhalation of yuzu fragrance and water in the follicular and late-luteal phases by a 22-year-old subject. LF: low frequency power (0.03–0.15 Hz); HF: high frequency power (0.15–0.5 Hz)

Figure 1 represents the case of ECG R-R interval changes and the corresponding power spectra before, immediately after, and 30 min after the 10-min aroma inhalation by a 22-year-old subject. In the follicular phase, the HF power representing the parasympathetic nervous system activity markedly increased right after the inhalation period with the yuzu fragrance. At 30 min after the inhalation period, the HF power slightly decreased but remained higher compared to the baseline. In the late-luteal phase, the HF power also apparently increased immediately after the inhalation period with the fragrance of yuzu and further increased at 30 min after the aroma inhalation period. Regardless of menstrual cycles, in contrast, the HF power did not change during the control trials with water.

**Fig. 2:**
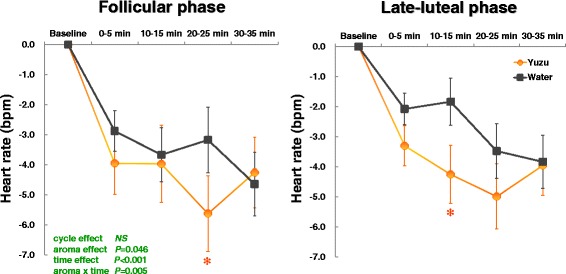
Changes of heart rates before (baseline) and after (0–35 min) the 10-min inhalation of yuzu fragrance and water in the follicular and late-luteal phases. A significant difference by paired *t* test was apparent between yuzu and control (water) trials (* *P* < 0.05)

Figure 2 shows the changes of heart rates after the aroma stimulations. We found no significant effects of menstrual cycle and the related interactions (cycle x aroma, cycle x time, cycle x aroma x time) on the change of heart rates. As the figure represents, heart rates more significantly decreased after the aroma stimulations with yuzu compared to water [aroma effect: *F*(1,16) = 4.70, *P* = 0.046; time effect: *F*(4,64) = 21.61, *P* < 0.0001; aroma x time effect: *F*(4,64) = 4.14, *P* = 0.005]. Further statistical analysis with paired *t* test revealed that heart rate more significantly decreased at 20–25 min in the follicular phase (*P* = 0.011) and at 10–15 min in the late-luteal phase (*P* = 0.045) after the 10- min inhalation of yuzu fragrance compared to water.

**Fig. 3:**
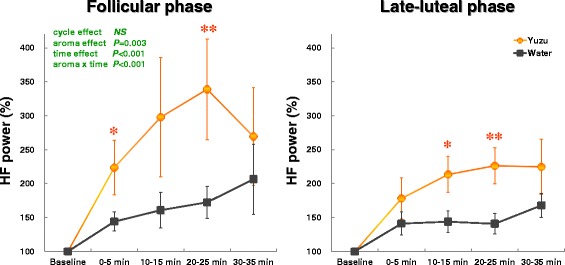
Rate of increase of high frequency (HF) power before (baseline) and after (0–35 min) the 10-min inhalation of yuzu fragrance and water. A significant difference by paired *t* test was apparent between yuzu and control (water) trials (** *P* < 0.01, * *P* < 0.05)

Figure 3 graphs the time course of the changes of HF power for 35 min after the aroma stimulations. After inhaling the yuzu aroma, the rate of increase in HF power seemed greater in the follicular phase. As with the change of heart rates, however, we found no effects of menstrual-cycle and the related interactions (cycle x aroma, cycle x time, cycle x aroma x time) on the change of HF power. We observed a significant increase in the HF power after the inhalation period of the yuzu scent in comparison with inhalation of the water [aroma effect: *F*(1,16) = 12.37, *P* = 0.003; time effect: *F*(4,64) = 9.43, *P* < 0.0001; aroma x time effect: *F*(4,64) = 5.79, *P* < 0.0001]. Paired *t* test further clarified that the rate of increase of HF power was greater at 0–5 min (*P* = 0.03) and 20–25 min (*P* = 0.008) in the yuzu trial compared to the control trial with water in the follicular phase. In the late-luteal phase, HF power increased more significantly at 10–15 min (*P* = 0.032) and 20–25 min (*P* = 0.005) in the yuzu trial than in the control trial with water. We also calculated the ratio of LF power to HF power indicating sympathovagal balance. As suggested previously [7, 8], however, no statistically significant difference was detected in the relative values between the two trials or the two menstrual phases.

### Changes of mood states 30 min after the inhalation period with yuzu fragrance

We found no significant menstrual-cycle effects and the interaction (cycle x aroma) on the TMD scores of the POMS test after the 10-min aroma stimulations. The statistical procedure, however, revealed significant aroma effects on the TMD [aroma effect *F*(1,17) = 5.86, *P* = 0.027]: The scores significantly decreased in the yuzu trials (follicular phase −8.4 ± 3.4, late-luteal phase −8.2 ± 3.2) compared to the control trial with water (follicular phase −1.8 ± 2.6, late-luteal phase 0.6 ± 1.4).

**Fig. 4:**
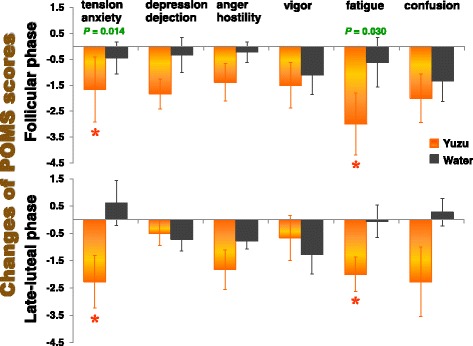
Subscore changes in the Profile of Mood States (POMS) test performed before and 35 min after the 10-min inhalation of yuzu fragrance and water. A significant difference was apparent between yuzu and control (water) trials (* *P* < 0.05)

Figure 4 illustrates the changes of the POMS subscales 30 min after the 10-min aroma inhalation period. As with the TMD scores, we found no significant menstrual-cycle effects and the interaction (cycle x aroma) on the six subscales of the POMS test. As to the aroma effect, in contrast, the subscores of tension–anxiety [aroma effect *F*(1,17) = 7.53, *P* = 0.014] and fatigue [aroma effect *F*(1,17) = 5.60, *P* = 0.030] significantly decreased after the inhalation of yuzu as compared to those of the control trial with water. Other negative symptoms—anger–hostility and confusion—also decreased more in the yuzu trial in both menstrual cycles, but the changes of the subscores did not statistically differ between the yuzu and control trials.

## Discussion

Yuzu, originally found in the upper reaches of the Yangtze River in China, arrived in Japan during the Sui Dynasty or Tang Dynasty over 1000 years ago [5]. The citrus fruits have been widely cultivated in Japan and have many uses, from cuisine and food products to health-promoting items. Yuzu has attracted worldwide attention: a 2003 article in the *New York Times* proclaimed yuzu the darling of chefs and as having an amazing floral citrus aroma [35]. The question emerges, as a matter of course, how and why does the yuzu aroma produce psychodynamic effects? To what extent does the autonomic nervous system, which plays a critical part in the integrity of the mind-body connection as the functional driver of general health and wellness, contribute to efficacious outcomes brought about by the fragrance?

The present study investigated the soothing effects of olfactory stimulation by the yuzu fragrance on autonomic nervous system function using HRV power spectral analysis in conjunction with psychoemotional symptoms among reproductive-age women. The study also explored the influence of menstrual phases on the aromatic effects of yuzu. The main findings reveal that only a 10-min inhalation of the yuzu scent significantly decreased heart rate and increased HF power of HRV reflecting parasympathetic nervous system activity, regardless of menstrual phases. This significant physiological effect continued for at least 25 min. POMS tests, in addition, demonstrated that inhalation of the aromatic yuzu oil significantly decreased TMD, a global measure of affective state, together with two POMS subscales—tension-anxiety and fatigue, as long as 35 min after the aroma stimulation, both in the follicular and late-luteal phases.

While we mentioned the paucity of information on the effects of yuzu inhalation, Kumagai et al. [36] reported their findings from an animal study in a Japanese aroma research journal: olfactory stimulation by the yuzu scent decreased activities of sympathetic nerves innervating brown adipose tissue and white adipose tissue in urethane-anesthetized rat. We found two other articles [7, 8] investigating the efficacy of yuzu aroma on human subjects, which were also published in Japanese scientific journals. A clinical study by Sawamura and colleagues [7] revealed that after inhaling yuzu fragrance (n = 24), an inpatient group fell asleep more easily on the night before an operation and woke up feeling better as compared to the control group (n = 26). These researchers also conducted an experiment to evaluate the physiological effects of one-minute olfactory stimulation from the yuzu fragrance by using ECG among healthy individuals (n = 13, 20–56 years [the sex ratio was not reported]). The results showed that heart rate significantly decreased after inhalation of yuzu aroma compared to the control trial with no fragrance. The coefficient of variation of R-R intervals, an index of parasympathetic nervous system activity, was greater in the yuzu trial than in the control trial, but the difference between the two trials was not statistically significant. According to Konno [8], who examined subjects with subclinical depression (male n = 4, female n = 4, 22.3 ± 2.8 years), a 7-min inhalation of yuzu fragrance significantly decreased two subscores of the short version of POMS—anger–hostility and fatigue—and increased the subscore of vigor. As to physiological measurements using acceleration plethysmography, the ratio of LF to HF power decreased, and the coefficient of the variation of the a-a intervals, indicating parasympathetic nervous system activity, increased, although the changes did not reach statistical significance.

Based on a literature survey using the PubMed database, as of March 1, 2016, the authors found 33 articles when using the keyword *yuzu* for the search. Only two articles, however, addressed human-subject studies to evaluate the soothing effects of yuzu aroma [6, 37]. From the psychological point of view, a clinical study with 121 participants (aromatherapy group n = 61, control group n = 60) conducted by Ueki et al. [37] showed that aromatherapy with yuzu fragrance significantly ameliorated the anxiety of mothers of sick children undergoing an infusion at a pediatric clinic. This study did not measure any physiological data including HRV measurements. The other article with a randomized controlled crossover study [6] demonstrated that 10-min inhalation of yuzu scent significantly decreased salivary chromogranin A—an endocrinological stress marker reflecting sympathetic nervous system activity—among healthy women (n = 20, 20.5 ± 0.1 year) in the follicular phase. In addition, the subscores of tension-anxiety, depression-dejection, anger-hostility, and confusion, as well as TMD, of the POMS test significantly decreased after the olfactory stimulation of the yuzu fragrance.

As to the psychological evaluation of yuzu aroma, the present study, as well as the earlier human studies mentioned above [6, 8], used the POMS test. All three investigations revealed that even a short-term inhalation of the yuzu fragrance decreased the POMS score of negative symptoms, indicating a healthcare intervention with yuzu aroma could improve emotional mood states. The results were, however, not always consistent among the studies. The differences in experimental designs and conditions, intensity of olfactory stimulation with yuzu aroma and clinical features of subjects might lead to the inconsistencies among the outcomes of the psychological measures.

When essential oils are inhaled, the individual volatile molecules are carried by eddy currents to the roof of the nose, where delicate cilia protrude from the receptor cells into the nose itself. When the molecules lock on to these hairs, an electrochemical message is transmitted via the olfactory bulb and olfactory tract to the primary olfactory regions in the brain, and most of the brain regions strongly relate to, or make up, part of the limbic system, the center of autonomic function and emotion [3]. The HRV power spectral analysis used in the present study serves as a valuable noninvasive device to evaluate sympathovagal activity. However, we should state that the device has a disadvantage: it cannot single out sympathetic nervous system activity, although some studies have referred to absolute or relative values of LF power or the LF to HF ratio as an alternative index of sympathetic nervous function [18, 22]. At the moment, we cannot fully elucidate the detailed mechanism of yuzu’s efficacy. However, the significant increase in HF power after the inhalation of yuzu found in the present study additionally suggests that yuzu interacts, at least in part, with the autonomic nervous system to increase parasympathetic nerve activity, which consequently modulates the cluster of negative psychoemotional symptoms.

To further explore the potential efficacy of the yuzu fragrance, the authors reviewed the literature on scientific research analyzing psychophysiological and behavioral effects of other citrus fruit essential oils. Both animal and human studies demonstrated that fragrance inhalation of essential oil from grapefruit (*Citrus paradisi*) increased sympathetic nervous system activity [38–40]. Limonene, accounting for approximately 95 % of the volatile components of grapefruit, contributes to sympathetic stimulatory effects and subjective alertness [38, 39]. Interestingly, other citrus fruits consisting of less limonene have the opposite effect of grapefruit on autonomic function. Female patients who were exposed to ambient fragrance from sweet orange (*Citrus sinensis*) peel oil, made up of limonene (88.1 %), myrcene (3.77 %), and α-pinene (1.19 %), showed a lower level of anxiety, a more positive mood, and a higher level of calmness in a dental office setting [41]. Three studies [22–24] evaluated the efficacies of bergamot essential oil (*Citrus bergamia*) by using HRV power spectral analysis and demonstrated that short-term inhalation of the fragrance generated from a diffuser significantly increased HF power. The results of the LF to HF ratios were inconsistent among the three studies. An investigation by Watanabe et al. [24] used bergamot essential oil, consisting of limonene (45.45 %), linalyl acetate (23.10 %), γ-terpinene (8.05 %), β-pinene (7.25 %), linalool (6.50 %), and α-pinene (1.35 %) and further clarified that salivary cortisol levels together with negative emotions, including anxiety, significantly decreased after inhaling bergamot fragrance. As we mentioned in Method, limonene is a major component (78.02 %) of the yuzu essential oil presently used, but the yuzu essential oil contains less limonene than the grapefruit essential oil. The second major component of yuzu, γ-terpinene (9.32 %), promotes dopamine release, resulting in stress reduction [42]. Yuzu shares other common components with lavender, a representative of relaxing essential oils, such as β-caryophyllene and linalool, which also have sedative effects [19, 21]. Yuzu, as well as sweet orange and bergamot, contains α-pinene—a major phytoncide, and has calming effects on autonomic stress response to novel environments [43]. A combination of limonene with these volatile components might augment parasympathetic nervous system activity to modulate the psychoemotional status. However, revealing the psychological, neurophysiological, and pharmacological functions of yuzu essential oil will require further interdisciplinary scrutiny and research.

The present study demonstrated no significant difference in the psychophysiological effects of the yuzu aroma during the menstrual cycle. The authors suggest caution in interpreting the findings since the data of the present study were obtained at two different time points, the follicular and the late-luteal phases. The influence of the menstrual cycle on olfactory sensitivity has been studied for over 50 years, but continues to be a matter of debate. For instance, according to a study by Doty et al. [44], peaks in olfactory sensitivity to the odor of furfural, a colorless liquid with a distinctive smell, were observed mid-cycle, mid-luteally, and during the second half of menses in women both taking and not taking oral contraceptives. In contrast, some investigators reported no menstrual cycle-related fluctuations in sensitivity to phenyl ethanol, androstenone, nicotine, and citral [45, 46]. Previous practical or clinical research revealed that aromatherapy alleviates menstrual-related problems, such as dysmenorrhea [47] and PMS [21, 48]. At the time of writing, however, no studies have examined whether the soothing effects of olfactory stimulation from fragrances or the efficacy of aromatherapy change during the menstrual cycle. To scrutinize the influence of the menstrual cycle on yuzu’s aromatic effects, future studies should be designed to measure a broader range of psychoneuroendocrinological parameters, including gonadal hormones and basal body temperature in addition to the autonomic functions and psychometric tools presently used, and to investigate them more frequently, i.e., menstrual, follicular, ovulatory, and early- and late-luteal phases, during a menstrual cycle among eumenorrheic women without oral contraceptives (OCs) and women with OCs as a control group. The authors’ previous studies demonstrated that the HF power of HRV at rest decreased in the late-luteal phase among women suffering from PMS, but women with no or slight premenstrual symptoms, categorized as premenstrual molimina, showed no menstrual cyclicity of autonomic nervous system activity [14, 15]. From the clinical point of view, therefore, we need to further investigate if the therapeutic efficacy of yuzu differs among women with different degrees of menstrual-related symptoms.

Several limitations of the present study deserve mention. First, the present study used two kinds of aromas, yuzu and water as a control. To avoid placebo effects, we did not inform subjects of which fragrance we would use for the experiments. We mentioned neither the concentration of the fragrance nor the use of water as a control trial. We cannot, however, completely deny the possibility that the participants would have noticed the difference when they inhaled the aroma of water. Second, as Table 3 shows, participants in this study evaluated the yuzu fragrance as pleasant. This study, however, employed a relatively small sample size. We thus need to explore the physiological response to the inhalation of yuzu essential oil among a larger sampling of women who consider the fragrance pleasant or unpleasant and compare the results between the two groups. To better explore the net psychological and pharmacological effects of the yuzu fragrance, it would be of interest to investigate experimental conditions with four olfactory stimuli—unscented water, the yuzu scent, and other kinds of scents considered both pleasant and unpleasant by participants. Finally, to scrutinize the efficacy of the yuzu aroma, further studies should be conducted to examine if the yuzu fragrance has gender-dependent effects on improving psychoemotional states. In addition, other factors, such as age and ethnicity, may influence olfactory and psychoneuroendocrinologic systems in humans.

## Conclusions

In conclusion, the present study, using HRV power spectral analysis, demonstrated that short-term inhalation of fragrance from yuzu essential oil significantly decreased heart rate and increased the HF power of HRV, regardless of menstrual phases. Although the underlying mechanisms of the soothing effects of yuzu remain unclear, this study indicates that yuzu’s aromatic effects could alleviate negative emotional stress, which, at least in part, contributes to the improvement of parasympathetic nervous system activity.
